# Exploring the Impacts of Urban Community Leisure on Subjective Well-Being during COVID-19: A Mixed Methods Case Study

**DOI:** 10.3390/ijerph19148514

**Published:** 2022-07-12

**Authors:** Jinwei Wang, Liyan Zhang, Yue Sun, Guangjuan Lu, Yanbin Chen, Saiyin Zhang

**Affiliations:** 1 Institute of Geographic Sciences and Natural Resources Research, Chinese Academy of Sciences, 11A, Datun Road, Chaoyang District, Beijing 100101, China; wangjinwei@bisu.edu.cn; 2 School of Tourism Sciences, Beijing International Studies University, No.1 Dingfuzhuang Nanli, Chaoyang District, Beijing 100024, China; 2021110281@stu.bisu.edu.cn (Y.S.); 2019110213@stu.bisu.edu.cn (G.L.); 3 Research Center for Beijing Tourism Development, No.1 Dingfuzhuang Nanli, Chaoyang District, Beijing 100024, China; 4 MTA/MBA Education Center, Beijing International Studies University, No.1 Dingfuzhuang Nanli, Chaoyang District, Beijing 100024, China; 2020110651@stu.bisu.edu.cn; 5 College of Geography and Environment, Shandong Normal University, No.1 Daxue Road, University Science Park, Changqing District, Jinan 250358, China; ychen@sdnu.edu.cn; 6 School of Statistics, Capital University of Economics and Business, Flower-Town, Fengtai District, Beijing 100070, China

**Keywords:** community leisure, subjective well-being, community identity, leisure satisfaction, leisure environment, COVID-19

## Abstract

Participating in community leisure activities has become an important way for the public to pursue good health and a high quality of life. However, few studies have focused on the health and welfare effects of participating in urban leisure activities during the COVID-19 pandemic. In response to this gap in the literature, this study drew on the stimulus–organism–response (SOR) theory to examine the effects of community leisure on subjective well-being during the pandemic from the perspective of urban residents. A sample of 1041 urban residents in Beijing, China, was empirically analyzed by applying partial least squares structural equation modeling (PLS-SEM) and fuzzy set qualitative comparative analysis (fsQCA). The PLS-SEM results revealed the net effects of the pandemic influence, leisure satisfaction, leisure environment, and community identity on subjective well-being. The fsQCA provided causal methods for realizing subjective well-being and a necessary condition analysis supplemented the necessary antecedents. The results showed that (1) higher levels of pandemic influence and leisure environment increased leisure satisfaction; (2) pandemic influence, leisure environment, and leisure satisfaction had a positive effect on community identity; (3) pandemic influence, leisure satisfaction, and community identity also combined with leisure environment and leisure time to positively influence subjective well-being; and (4) leisure satisfaction and community identity mediated the impacts of pandemic influence and leisure environment on subjective well-being. This paper contributes not only to empirical evidence but also to theory by constructing and enriching the research models of community leisure and subjective well-being. The practical implications for the public, community managers, and policymakers are also discussed.

## 1. Introduction

The COVID-19 pandemic has swept the world since 2020 and has disturbed normal life and work for most people. Many countries have implemented a series of measures, such as lockdowns and the isolation of individuals and families, to deal with the crisis. The pandemic has psychologically and physically isolated people by limiting physical exercise and human interaction; many people have had to study and work from home and had their movements restricted, including their ability to travel. In addition, the pandemic has had a significant impact on the world economy, including an increase in market uncertainty and an erosion of investor confidence, which has put huge pressure on economic operations from both the consumption and production sides [1]. During this time, people have become increasingly aware of the significance of leisure activities.

“Community leisure” is the general term for all activities that community residents carry out within the community during their leisure time. It can involve the use of community facilities to relax the body and mind and perform recreational health activities [2]. Approaching leisure from the perspectives of residents and communities, Cohen-Gewerc [2] noted that moderate leisure can result in physical and mental pleasure and satisfaction and is an important way for human beings to relax and improve their health. Therefore, residents can gain a sense of pleasure from leisure activities, thus forming a sense of pride and community identity. From the perspective of society, community leisure activities significantly improve community support and social cohesion [3]. From the perspective of urban economic and social development, community leisure provides many functions, such as recreational, sportive, cultural, and tourist functions [4]. In this paper, “community leisure” refers to a series of activities (e.g., social and sportive activities) that are conducted with family and friends within urban communities for relaxation and health outside of working hours [5].

“Subjective well-being” (SWB) is an individual’s overall evaluation of their life quality according to self-defined standards and is used to describe the happiness level that that individual experiences [6]. High levels of SWB are associated with better health and longevity [7] and SWB has been shown to play a positive role in promoting the harmonious and stable development of society [8]. From a variety of theoretical perspectives, researchers have found that improvements in SWB are conducive to improving the physical and psychological health of individuals [9], increasing their income and labor productivity [7], and promoting trust and social harmony [7]. Understanding the SWB of individuals can also promote the implementation of targeted strategies to improve their overall happiness.

Therefore, to promote the development of community leisure in the context of the COVID-19 pandemic, we developed a model to examine the relationships between com-munity leisure, community identity, and subjective well-being. Overall, the study was driven by two main goals: (1) to examine the relationships between community leisure, community identity, and subjective well-being and (2) to find effective ways to promote the development of leisure within the context of the COVID-19 pandemic. Based on the stimuli–organism–response (SOR) theory, this study applied a combination of PLS-SEM and fsQCA techniques to explore these objectives. The results contribute to existing understanding of the relationships between community leisure and subjective well-being, which inform best practices for community development that is geared toward improving the happiness of residents.

## 2. Literature Review, Conceptual Framework, and Hypotheses

### 2.1. The Stimulus–Organism–Response (SOR) Framework

The SOR framework was established by Mehrabian and Russell in 1974 to explain how people produce corresponding behavioral responses under the stimulation of different external environmental factors and was based on three elements: a stimulus, an organism, and a response [10]. The first component of “stimulus” refers to the state of an individual being affected due to an external influence. The second element of “organism” captures the individual’s cognitive and affective state, which is in turn reflected in the intermediate steps that are taken between the stimuli and the response. The outcome of the SOR framework is the individual’s approach or avoidance behavior [11]. The SOR framework establishes that an individual’s exposure to external stimuli (S) creates his or her internal state (O), which triggers his or her response (R) [10]. Generally, researchers have applied this model to study consumer purchasing behavior and online user information behavior [11].

However, some scholars have applied the SOR theory to carry out research within the field of community leisure. For example, Sohaib et al. [11] used the SOR theory to explore the impacts of nature-based solutions on the mental health, emotional well-being, brand attitudes, and green brand loyalty of people within the hotel community. Meanwhile, Loureiro et al. [12] used the SOR paradigm to help them to uncover the atmospheric cues and involvement that are important antecedents to relaxation and pleasure. For the purposes of this paper, it is important to note that the SOR theory can effectively explain the relationships between external stimuli, bodily perception, and responses during community leisure activities. Therefore, we used the SOR framework to construct a research model, in which we considered the pandemic influence (i.e., the impacts of the COVID-19 outbreak on lifestyle) and the leisure environment as the stimuli, leisure satisfaction and community identity as the organisms, and subjective well-being as the response.

### 2.2. Community Leisure

“Leisure” refers to a variety of non-working activities that individuals engage in during their leisure time. It is a common and normal human behavior and can be a site of social construction and human personality development [13]. After the industrial revolution, Marx proposed that the implications of the value of leisure and the free and comprehensive development of human beings should be integrated into monism through the investigation and study of the working class, which laid the foundations for the birth of leisure science (which is generally regarded as beginning in 1899 with Veblen’s *Theory of the Leisure Class* [14]. Later scholars explored leisure from the perspectives of philosophy, society, economy, education, and psychology, among other fields, and discussed the status of leisure throughout the history of human thought, the relationships between leisure and life existence, and the essence of leisure freedom in multiple dimensions. They emphasized the important role of leisure and aesthetic education in life creation and the peak experience of leisure aesthetics [15].

Leisure research remains an active field, especially for elderly and vulnerable groups. Leisure purposes and environmental factors impact leisure characteristics, site selection, and related tourism behaviors [16]. From a disciplinary point of view, leisure studies have been widely used in psychology, sociology, and other disciplines. For instance, Cho and Chiu [17] studied the relationships between leisure centrality, self-satisfaction, and academic intrinsic motivation among college students. Moreover, Crandall and Thompson [18] confirmed the importance of social interaction in cross-cultural leisure, which laid the foundations for future research on the influences of different factors on the relationship between social interaction and leisure.

Against this background of the deepening of leisure research, although the concept of community leisure research was formed later on, it has received attention from many experts and scholars. Early studies on community leisure have mainly focused on the design of community leisure activities, the application of leisure facilities, and the impacts of leisure activities on intra-community relationships. For example, Balogun [19] established critical links between leisure activities, recreation facilities, and the built environment on one hand and community health on the other hand. Over recent years, leisure scholars have paid increasing attention to the social attributes of communities, believing that the community is an important space for studying cultural and social integration within a leisure context.

Thus, community leisure has become an important field of leisure research. From the perspective of residents within a given community, the development of community leisure activities can contribute to the development of the physical and mental health of the residents [20]. Studies have shown that physical activity levels are affected by a variety of factors, such as resident health status, public policies, the social system, and the exercise environment, among which environmental factors are particularly important [16]. Notably, Balogun [19] found that leisure activities and recreational facilities are substantial factors in promoting community health. From the perspective of society, community leisure activities can promote community identity and thus, promote the harmonious and orderly development of society. Morata et al. [21] confirmed that community leisure activities have a significant effect on improving community support and social cohesion.

### 2.3. Subjective Well-Being

Subjective well-being is usually used to describe the happiness level that is experienced by people based on the subjective evaluation of their lives [6]. As a psychological indicator, the measurement of subjective well-being is not simple and there are many different academic opinions about the measurement scale of subjective well-being. Representative measurement methods mainly include the life satisfaction evaluation method and the hedonic well-being measurement method [22]. Diener and Chan [7] discussed subjective well-being from different theoretical perspectives and confirmed that subjective well-being has a positive impact on people’s lives. Improvements in subjective well-being are conducive to increasing resident income, labor input, and labor productivity, maintaining political stability, reducing crime rates, improving resident health, reducing divorce rates, and enhancing social harmony and the trust between people.

The research on factors that influence subjective well-being has been an important part of the leisure research field. Income has long been considered to be an important factor in improving people’s happiness, but Easterlin [23] confirmed that while economic growth is positively correlated with happiness in the short term, this positive correlation disappears in the long term in what is called “the Easterlin paradox”. In addition, gender, age, ethnicity, education, and other personal characteristics, as well as society, politics, and economy, all have impacts on subjective well-being [24]. Regarding social characteristics, studies have been conducted on the relationships between economic growth, unemployment, inflation and happiness, income gaps, inequality and happiness, social relations, social status, and public security and happiness [25].

A comprehensive survey of empirical studies on the relationship between leisure and subjective well-being revealed that existing research has had three main foci. First, exploring the effects of different leisure activities on subjective well-being. Studies have pointed out that different types of leisure activities can improve subjective well-being, such as sports and social contact [16]. Compared to shallow leisure, individuals can obtain higher levels of satisfaction and happiness from deep leisure [26]. Second, studying the differences between different groups taking part in leisure activities and their happiness; for instance, there have been studies on adolescents [16] and the elderly [17]. Third, analyzing the mechanism of interaction between leisure and subjective well-being from the perspectives of individual demand and leisure motivation [6]. Therefore, a good deal of work has been carried out on the subjects (the people engaging in leisure activities) and objects (the kinds of leisure activities in which they are engaging) of leisure activities, but little work has been conducted on the impacts of community leisure on subjective well-being.

Meanwhile, scholars have recently been deepening their research on subjective well-being and have produced some fruitful results. Although, as the above review reveals, many studies have been conducted on the factors that influence subjective well-being, relatively few studies have been carried out on the relationship between leisure and subjective well-being. To enrich knowledge on the latter topic, this study explored the factors that influence subjective well-being and the relationship between subjective well-being and community leisure using a case study of Beijing residents and structural equation modeling. Ultimately, this study sought to provide a new perspective on the development of subjective well-being at the community resident level and to serve as a reference for community leisure management.

### 2.4. Research Model and Hypothesis Development

**Figure 1:**
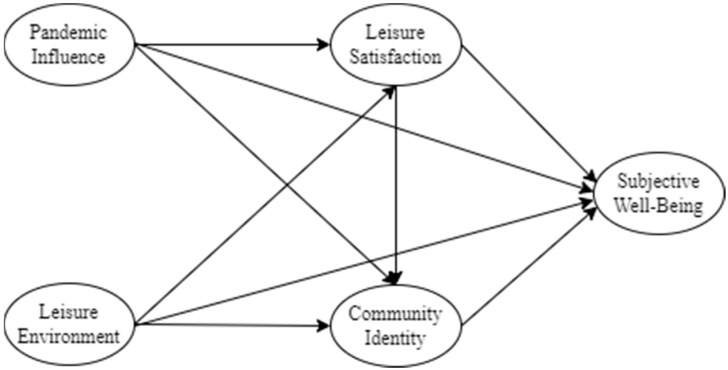
Our conceptual model.

According to the SOR theory and the above literature review, this study took pandemic influence and leisure environment as the external stimuli (S), leisure satisfaction and community identity as the organic factors (O), and subjective well-being as the reflection factor (R) to establish a structural model (Figure 1). The model consisted of 5 structural variables and 11 hypotheses.

All of our hypotheses are detailed below.

(1) Factors that influence leisure satisfaction.

“Leisure satisfaction” refers to the positive feelings that are displayed, formed or obtained by individuals due to their participation in leisure activities and the degree of satisfaction that they feel from the leisure experience [27]. After the COVID-19 outbreak, urban community residents began to pay much more attention to the different elements of leisure, such as leisure time, leisure activities, and leisure environments [28]. During the pandemic, leisure activities, especially sports and exercise activities, were associated with positive emotions and high levels of life satisfaction, in addition to good physical health [29]. Therefore, the pandemic influence may play a positive role in leisure satisfaction through a particular mechanism. Accordingly, we proposed the following hypothesis:

**H1:**  *Pandemic influence is positively related to leisure satisfaction.*

Elements such as community greening, community space, and community services together constitute the community leisure environment [30]. A good leisure environment can have positive impacts on urban community residents in terms of physical health, social communication, identity, and belonging [31]. Along these lines, Weliange et al. [32] found that high-condition environments can enhance neighborhood harmony. Meanwhile, Saloma et al. [33] confirmed that public green spaces are more conducive to fostering a sense of community and metropolitan identity than high-condition environments. Therefore, we reasoned that a comfortable leisure environment is likely to provide residents with a strong sense of leisure, thus improving their leisure satisfaction. We consequently postulated that:

**H2:**  *Leisure environment is positively related to leisure satisfaction.*

(2) Factors that influence community identity.

“Community identity” refers to the sense of identity of the residents within a community. It reflects the recognition of community functions by residents and the intensity of their emotional connection to the community [34], which can enhance their sense of belonging and pride. As mentioned above, the pandemic has positively influenced most people to some extent as residents have become more active in community leisure activities and have enjoyed higher levels of leisure satisfaction. Identity theory is a research perspective from which individual social behavior can be explained according to the interactions between an individual’s cognition and a social mechanism. Because this can connect the individual characteristics of participants with a local social structure, it can serve as a theoretical tool for analyzing the logic behind resident participation within urban communities. Therefore, it can help to clarify the mechanism of interaction between resident sense of identity and community participation [35]. We consequently postulated that:

**H3:**  *Pandemic influence is positively related to community identity.*

The leisure environment is of great significance to the development of community leisure activities. A positive leisure environment provides a good and comfortable leisure experience for community residents, thereby affording residents a strong sense of responsibility as community owners. Ultimately, this can help residents to form a strong sense of community identity. Existing studies have confirmed that place attachment plays a mediating role in the relationship between outdoor leisure environments and well-being [36]. Moreover, the perception of the environment and physical activity during leisure time also contribute to the formation of community identity [37]. Therefore, we concluded that there is likely a positive relationship between leisure environment and community identity. We consequently postulated that:

**H4:**  *Leisure environment is positively related to community identity.*

Further, leisure satisfaction can reflect the degree to which community residents are satisfied with their community leisure activities. Community identity is an important factor that drives the relevant behaviors of community residents [35]. The satisfaction that can be gained from community leisure helps to enhance the recognition of community functions by residents and strengthen their emotional connection to the community, which positively impacts community identity. Some scholars have further explored this phenomenon. For example, Arfini et al. [38] innovatively studied the construction process of community identity, taking the online community as an example, and proposed specific approaches to the construction of community identity based on their results. Kokab et al. [39] argued that community identity can promote the wider integration of communities in different locations to meet health and lifestyle needs. These studies suggested that there is a positive relationship between leisure satisfaction and community identity. In order to explore the specific mechanisms between these factors in the terms of community leisure, we proposed the following hypothesis:

**H5:**  *Leisure satisfaction is positively related to community identity.*

(3) Factors that influence subjective well-being.

Subjective well-being involves a multidimensional evaluation of life, including the cognitive judgment of life satisfaction and the emotional evaluation of feelings and emotions [6]. Under the positive influence of the pandemic, urban community residents have been more likely to devote themselves to leisure activities, thus improving their leisure satisfaction and subjective well-being [28]. Through this mechanism, pandemic influence and leisure satisfaction have positive effects on subjective well-being. Notably, McCallum et al. [40] studied and verified this mechanism of action and Carrillo et al. [41] reported that there is an interaction between subjective well-being and leisure satisfaction. These studies suggested that this positive relationship does exist, but that the embodiment of the specific mechanisms within the field of community leisure needs further analysis and verification. Therefore, in order to further explore the relationships between pandemic influence, leisure satisfaction, and subjective well-being, we proposed the following hypotheses:

**H6:**  *Pandemic influence is positively related to subjective well-being.*

**H7:**  *Leisure satisfaction is positively related to on subjective well-being.*

As mentioned above, a good leisure environment provides better leisure experiences for community residents, enhances their sense of ownership, and forms a strong sense of community identity. Community identity involves the emotional attachment of residents to their community and reflects the consistency between the personal identities of the residents and their community environment in terms of aspects such as preferences, perceptions, and values [34]. Regarding the resident–community relationship, residents experience their environment by interacting with the community. The integration of personal identity into emotion is transformed into memories that are related to the community; this forms a resident’s sense of their community’s identity, which they internalize as a part of their own individual experience [35]. In other words, community identity builds the emotional and value-based link between community capability and community development, which can in turn enhance belonging and pride among community members and thus, enhance the subjective well-being of the community residents. The stronger the resident sense of community identity, the greater their confidence in and expectations for community development [20]. In fact, this can invisibly improve resident subjective well-being. Accordingly, we proposed the following hypotheses:

**H8:**  *Leisure environment is positively related to subjective well-being.*

**H9:**  *Community identity is positively related to subjective well-being.*

(4) The mediating effects of leisure satisfaction and community identity.

Leisure satisfaction is an important variable that affects leisure attitudes and life satisfaction [27] and the study of leisure satisfaction is of great significance to the improvement of the quality of life of urban residents. The positive impacts of the pandemic on community residents have made them enjoy leisure more and thus, has improved their leisure satisfaction and in turn their subjective well-being. Studies have found that participation in family leisure activities contributes positively to happiness [42]. Leisure satisfaction is the main predictor of family life satisfaction and core leisure activities are the main factors that influence family life satisfaction [43]. Kuo et al. [44] conducted research using the cognition–impact–behavior model and found that local identity plays a mediating role in the influences of social capital on support intention. It has been speculated that community identity may play a mediating role in the relationship between pandemic influence and subjective well-being. Therefore, the following hypothesis was proposed:

**H10:**  *Leisure satisfaction and community identity mediate the positive relationship between pandemic influence and subjective well-being.*

Notably, community identity plays an irreplaceable role in the construction and development of a harmonious community. The formation of community identity is realized through the process of helping to protect the cultural characteristics and collective memories of a living space [35]. For our purposes, it is important to note that the positive impact of the pandemic on community residents has enhanced their sense of community identity. However, “community identity” also describes the quality of the relationship between a tourist destination and its residents, which is an important factor for improved subjective well-being among urban community residents [34]. Within the field of community research, Harding [45] explored the importance of communication by studying how foreigners establish and maintain their communities. Community identity can improve the quality of life of residents and thus, has a positive effect on their well-being [46]. Therefore, we speculated that community identity may play a mediating role in the relationship between pandemic influence and subjective well-being. The following hypothesis was subsequently proposed:

**H11:**  *Leisure satisfaction and community identity mediate the positive relationship between leisure environment and subjective well-being.*

## 3. Samples and Methods

### 3.1. Study Site

**Figure 2:**
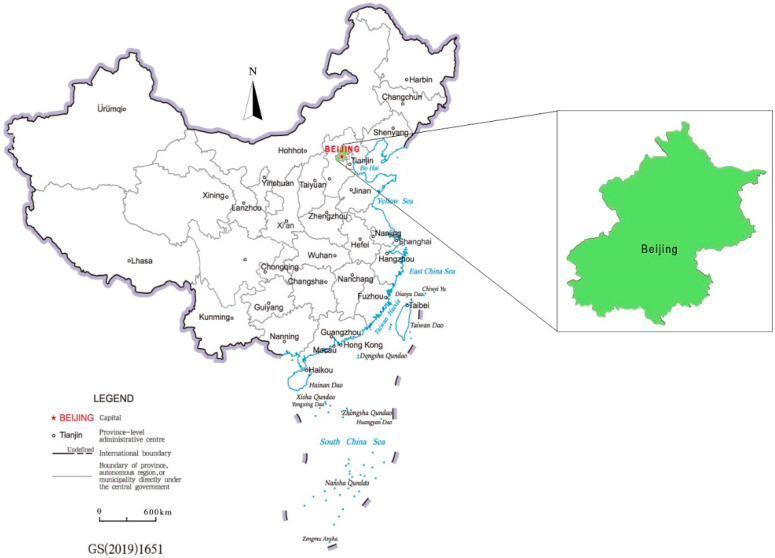
The location of Beijing, China.

**Figure 3:**
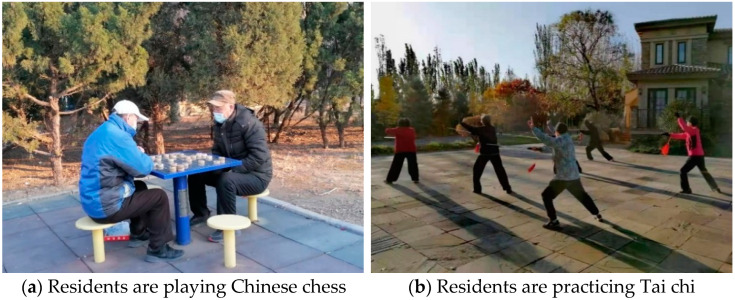
Residents participating in community leisure activities.

We chose Beijing, the capital of China, as the study site for the following reasons. First, Beijing (Figure 2) has taken extensive measures to strengthen the construction of its community leisure environments, create a community leisure atmosphere, and encourage residents to actively participate in community leisure activities (Figure 3) through programs such as the 15-Minute Service Circle and Green Community Creation. Second, Beijing is a megalopolis with a population of over 20 million people and has become a key city in China’s pandemic prevention and control regime; therefore, it can be used as a reference for other similar super-large cities in the context of the pandemic. Finally, it is a city with a large migrant population and as more people have returned to Beijing, the task of pandemic prevention and control has become more difficult.

### 3.2. Measures

A questionnaire was used to collect information from Beijing residents. The questionnaire contained six major sections. It was ultimately designed to reveal the potential linkages between pandemic influence, leisure environment, leisure satisfaction, community identity, and SWB. The items are listed in Table 1. Specifically, the questionnaire was organized as follows: (1) pandemic influence was assessed utilizing seven items from the scales of Wenham et al. [47] and Wong et al. [48]; (2) leisure environment was assessed utilizing five items from the scales of Burmeister et al. [49] and Pereira et al. [50]; (3) leisure satisfaction was assessed utilizing five items from the scales of Wang [51] and Dai et al. [52]; (4) community identity was assessed utilizing seven items from the scales of Thongpanya [53] and Bacon and Kaya [54]; (5) SWB was assessed utilizing seven items from the scales of Etxeberria [55] and Sumargo and Novalia [56]; and (6) further items were included to collect the demographic information of the participants, including age, gender, education level, and type of residence.

### 3.3. Data Collection

The participants were community residents in Beijing. A pre-survey was conducted using network and onsite random sampling on 20 May 2020. We adjusted the questionnaire based on the feedback we received about the content and phrasing of the questionnaire before finalizing it.

The questionnaire survey was conducted with the informed consent of the participants. From 29 May to 9 September 2020, questionnaires were distributed in two different ways: (1) an onsite survey, for which we randomly selected residents from communities within Beijing, informed them about the purpose of the survey through face-to-face communication, and asked them to fill in a paper questionnaire on site and (2) an online survey (which was used to reduce the risk of exposure to COVID-19), for which we sent a link to the questionnaire to Beijing residents via Tencent QQ and WeChat (the two most popular instant messaging apps in China), forwarded the link to WeChat moments (through which users can post text and pictures to express their thoughts), and used snowball sampling (random sampling seemed difficult within the pandemic context).

The questionnaires were mainly distributed in 13 districts of Beijing and a total of 1568 copies were collected. After excluding questionnaires that took less than an average of 102 s to complete and questionnaires that had the same answer for all questions, a total of 1041 valid questionnaires were obtained, with an effective rate of 66.4%.

### 3.4. Statistical Analysis

SPSS 26.0 software was used to analyze the possible common method deviations. Based on the exploratory nature of the study objectives and the non-normal sample distribution, PLS-SEM was selected to explore the complex mechanisms of community leisure [57]. Second, to analyze whether a single condition or a combination of conditions was sufficient or necessary to explain these mechanisms [58], we used fsQCA software 3.0 to conduct a univariate necessity analysis and a conditional configuration analysis on the basis of data calibration.

## 4. Empirical Analysis

### 4.1. Demographic Information

The demographic information of the participants is shown in Table 2. In terms of gender, females constituted 60.3% of the sample and males constituted 39.7%. Regarding age, 19.5% of the sample were between 20 and 30 years old and 27.7% were between 30 and 40 years old. Regarding education, most participants held a bachelor’s degree (42.7%) or a master’s degree or above (28.1%). In terms of occupation, 36.0% were enterprise staff and 15.7% were students. Regarding community type, most participants lived in commercial residential areas (48.6%). Regarding the length of residence, most participants (27.7%) had lived in their communities for 3 years or less.

### 4.2. The Structural Model Results

#### 4.2.1. Common Method Variance Analysis

The Herman’s single factor analysis method was used to check whether there was common method variance. The results showed that there were five factors with characteristic values that were greater than 1. The explanatory variation of the first factor was 47.933%; however, because it was less than 50%, there was no serious common method variance [59].

#### 4.2.2. Reliability and Validity Test

Anderson and Gerbing [60] advocated for two-step guidelines when analyzing a conceptual model, stating that the structural properties of a conceptualized model can only be tested when reliability and validity are confirmed. Therefore, we conducted reliability and validity tests (Table 3). The item loading, composite reliability (CR), average variance extracted (AVE), and Cronbach’s alpha values indicated that the results were reliable and valid. All outer loadings of the reflective constructs were well above the minimum threshold value of 0.622 and the constructs had high levels of internal consistency and reliability, according to the CR values. Furthermore, the AVE values were well above the minimum level of 0.50, implying the convergent validity of the research constructs. The Fornell and Larcker [61] criterion (Table 4), loading and cross-loading criterion, and HTMT (Table 5) showed the discriminant validity of all constructs. In all cases, an indicator loading on its own construct was higher than all of its cross-loadings with the other constructs. The HTMT ratio of correlation showed that all values were below the threshold of 0.85, thereby establishing the discriminant validity of the reflective constructs.

#### 4.2.3. Structural Model Analysis

**Figure 4:**
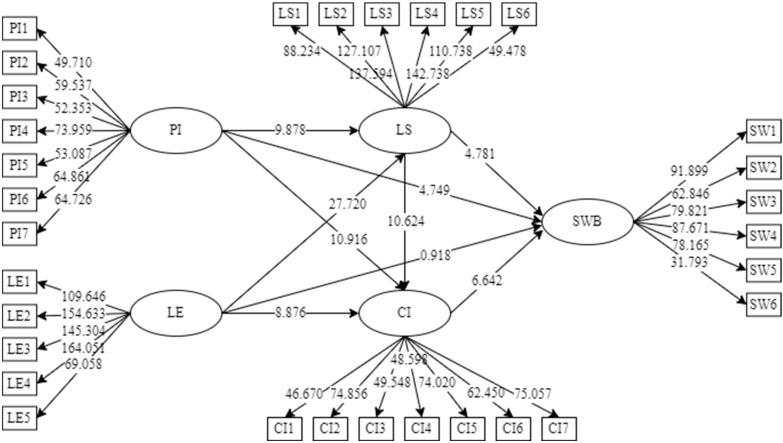
The bootstrapping results (empirical assessment using the PLS-SEM method). PI, pandemic influence; LS, leisure satisfaction; LE, leisure environment; CI, community identity; SWB, subjective well-being.

As is shown in Table 6 and Figure 4, pandemic influence had a positive effect on resident leisure satisfaction (*B* = 0.245, *t* = 9.960, and *p* < 0.05), community identity (*B* = 0.320, *t* = 10.971, and *p* < 0.05), and SWB (*B* = 0.180, *t* = 4.817, and *p* < 0.05). Hence, H1, H3, and H6 were supported.

Figure 4 shows that leisure environment had a positive effect on resident leisure satisfaction (*B* = 0.662, *t* = 27.650, and *p* < 0.05) and community identity (*B* = 0.291, *t* = 9.953, and *p* < 0.05). Thus, H2 and H4 were supported. In contrast, the results indicated that leisure environment did not influence SWB (*B* = 0.037, *t* = 0.933, and *p* > 0.05); therefore, H8 was not supported.

Similarly, leisure satisfaction had a positive effect on resident community identity (*B* = 0.355, *t* = 10.876, and *p* < 0.05) and SWB (*B* = 0.227, *t* = 4.790, and *p* < 0.05) and community identity also had a positive effect on resident SWB (*B* = 0.344, *t* = 6.723, and *p* < 0.05). Hence, H5, H7, and H9 were supported.

The mediating effects were analyzed using the Sobel test (Table 7). In the PI → SWB path, the VAF value was between 0.2 and 0.8, indicating the existence of a partial mediation effect. In the LE → SWB path, the VAF value was greater than 0.8, indicating the existence of a complete mediation effect. Therefore, H10 and H11 were supported.

### 4.3. The fsQCA Results

#### 4.3.1. Calibration

The original data needed to be calibrated before the qualitative comparative analysis could be performed. The direct calibration method was used to convert the data into fuzzy set membership scores and the three anchor points were defined as 95%, 50%, and 5%, according to the relevant literature [62]. The results are shown in Table 8.

#### 4.3.2. Univariate Necessity Analysis

A necessity test was then performed (Table 9). The consistency of all of the conditional variables of urban community resident SWB was less than 0.9; thus, it did not constitute a necessary condition [62]. Therefore, for the influential factors in this study, there was a need for further synergistic effects.

#### 4.3.3. Conditional Configuration Analysis

A configuration analysis was also conducted (Table 10). The consistency threshold was set at 0.8 and the frequency threshold was set at 1. Six configuration paths were obtained. The overall consistency was 0.7270 and the coverage rate was 0.9210, indicating that the six configurations could explain 92.1% of the cases with a good explanatory power.

**Figure 5:**
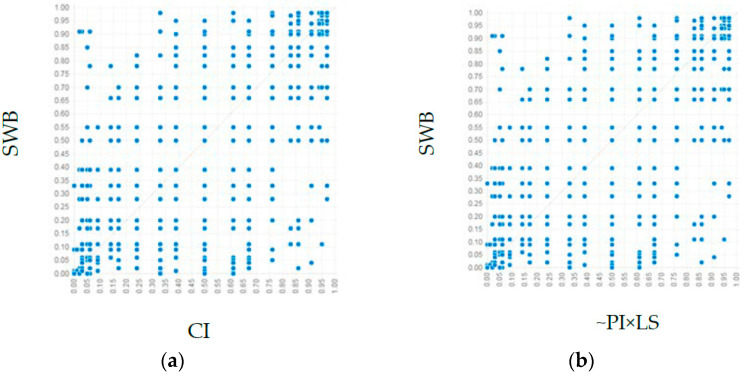

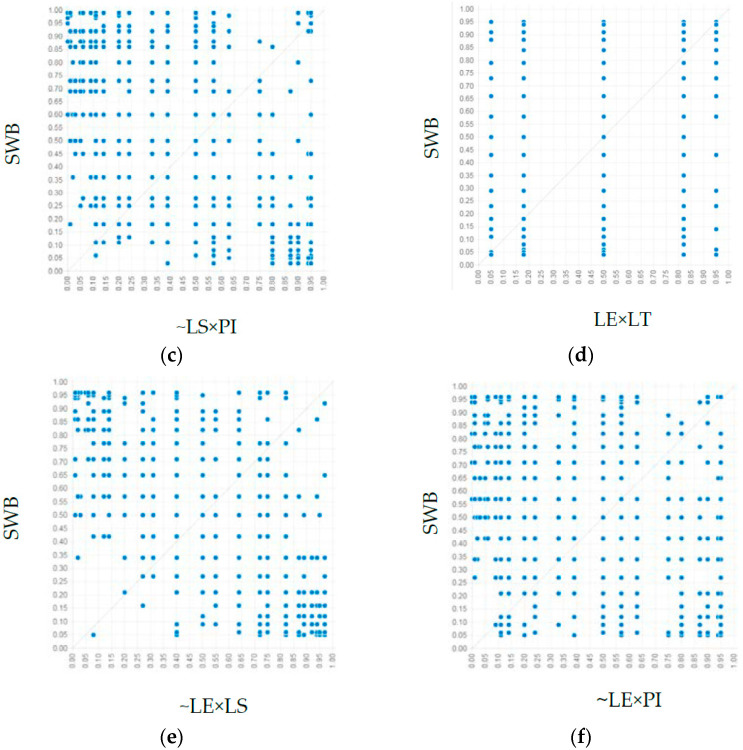
The XY plots of the solutions for sufficient conditions: (**a**) the distribution of configuration 1; (**b**) the distribution of configuration 2; (**c**) the distribution of configuration 3; (**d**) the distribution of configuration 4; (**e**) the distribution of configuration 5; (**f**) the distribution of configuration 6. PI, pandemic influence; LS, leisure satisfaction; LE, leisure environment; CI, community identity; SWB, subjective well-being.

As is shown in Table 10 and Figure 5, six configurations were produced and integrated into four possible solutions for high levels of SWB.

In solution 1, as shown in configuration 1, community identity was the core condition of a high level of SWB, which was consistent with H9, i.e., community identity alone as an influencing factor could be a sufficient condition for the outcome variable. This combination could explain 82% of the cases, whereas 7% of the cases could only be explained by this approach. Solution 1 indicated that when the community identity of urban residents was at a high level, even when their leisure satisfaction and other factors were at low levels, their SWB level could be high.

In solution 2, as shown in configurations 2 and 5, leisure satisfaction was a marginal condition of high SWB, which was consistent with H7. Configuration 2 showed that leisure satisfaction alone could be a sufficient condition for the outcome variable. This combination could explain 56% of cases, whereas 0.8% of cases could only be explained by configuration 2. Similarly, configuration 5 showed that leisure satisfaction could independently constitute a sufficient condition for high levels of SWB. This path could explain 48% of cases, whereas 0.04% of cases could only be explained by configuration 5. Solution 2 indicated that when the community identity of urban community residents was at a high level, their SWB levels could be high even when their leisure satisfaction and other factors were at low levels.

In solution 3, as shown in configurations 3 and 6, pandemic influence was a marginal condition of high SWB, which was consistent with H6. Configuration 3 showed that pandemic influence alone could be a sufficient condition for the outcome variable. This combination could explain 41% of cases, whereas 0.1% of cases could only be explained by configuration 3. Similarly, configuration 6 showed that pandemic influence alone could constitute a sufficient condition for high levels of SWB. This path could explain 45% of cases, whereas 0.07% of cases could only be explained by configuration 6. Solution 3 indicated that when pandemic influence was at a high level, even when community identity and other factors were not at high levels, resident SWB levels could be high.

In solution 4, as shown in configuration 4, leisure environment and leisure time were the core conditions of high levels of SWB, which further explained and complemented H8. Configuration 4 showed that leisure environment and leisure time together constituted sufficient conditions for the outcome variable. This path could explain 71% of cases, whereas 1.2% of cases could only be explained by configuration 4. Solution 4 indicated that the leisure environment level could be high even when community identity and other factors were at low levels.

## 5. Discussion

### 5.1. Findings

Through a comparative and integrated analysis of the results, we drew the following conclusions.

First, pandemic influence and leisure environment have a positive effect on leisure satisfaction. Such relationships have been observed in previous studies, which have confirmed that the pandemic indirectly improved leisure satisfaction by making people more attentive to and engaged in leisure activities [29]. Additionally, Li et al. [63] suggested that a sense of community is positively associated with street greenery at the community level. This study verified the relationship between these three variables within the same setting. Moreover, it also confirmed that COVID-19 impacted community leisure, in part by influencing resident leisure satisfaction. In the context of the pandemic, the impact of the leisure environment (which is an important part of community leisure) on resident leisure satisfaction cannot be ignored. Our findings inspired us to account for not only basic leisure components but also unexpected factors, such as COVID-19, when studying community leisure.

Second, pandemic influence, leisure environment, and leisure satisfaction have a positive effect on community identity. This finding aligned with prior studies, which have found that the perception of the environment contributes to the formation of community identity [37]. In addition, Arfini et al. [38] highlighted that a sense of self plays a role in what individuals care about, which shapes their affective life and normative worldview. Our first finding added to this literature by proving that pandemic influence, leisure environment, and leisure satisfaction can help residents to form community identity. However, this study also more specifically enriched existing knowledge by showing the influences of the COVID-19 pandemic and community leisure on community identity.

Third, pandemic influence, leisure environment, community identity, and the combination of leisure environment and leisure time have positive effects on SWB. Our results showed that pandemic influence, leisure satisfaction, and community identity affected resident SWB, but leisure environment alone had no significant influence on SWB. However, our fsQCA analysis revealed that the combination of leisure environment and leisure time had a positive effect on SWB. This finding was also consistent with existing studies. Dingle et al. [20] noted that residents with a strong identification with their community have an enhanced sense of SWB from their increased confidence in and expectations for community development. Ultimately, our findings offered insights into the realization paths of subjective well-being, especially within the context of community leisure, which may be useful for tourism research.

Fourth, leisure satisfaction and community identity partially and significantly mediate the relationship between pandemic influence and SWB and fully mediate the link between leisure environment and SWB. Previous studies have confirmed that the COVID-19 pandemic and built environments have an effect on SWB [64,65]. This study further found that given a higher level of leisure satisfaction and community identity, residents were more likely to have a strong sense of SWB in better leisure environments and under the positive pandemic influence.

### 5.2. Theoretical Implications

This study’s use of the SOR theory reflected the proposition that resident SWB is an objective behavioral response that is stimulated by pandemic influence and leisure environments through the media of leisure satisfaction and community identity. The theoretical contributions of this study are as follows.

First, this study enriched existing research on community leisure by taking the positive pandemic factor into consideration. The COVID-19 situation has affected individuals and communities across the world; however, considering its positive impact is conducive to promoting the long-term and stable development of community leisure.

Second, using the SOR theory, this study constructed and enriched the research model of community leisure and SWB. By exploring and analyzing the relationships between pandemic influence, leisure satisfaction, leisure environment, community identity, and SWB, this study proposed an explanatory framework for the formation of the relationship between community leisure and SWB, which could have theoretical implications for future research.

Third, given the complexity of resident community leisure, we combined PLS-SEM and fsQCA to better understand the formation of SWB. The PLS-SEM method revealed the net effects of different variables on SWB and the fsQCA method provided causal methods for realizing subjective well-being. By using a combination of methods, this study uncovered comprehensive evidence of the mediating effects of leisure satisfaction and community identity, which could be an interesting topic for future research.

### 5.3. Practical Implications

Our study has strong practical implications for the public, community managers, and policymakers.

First, the public should be made aware of the importance of community leisure and its effects on physical and mental health. The COVID-19 outbreak has increased our awareness of the importance of safety and physical health [66]. Community leisure activities can allow residents to engage in physical exercise and improve their health. At the same time, active participation in community leisure activities can delight the body and mind and promote the well-being of entire families. Leisure activities ultimately play a positive and irreplaceable role within community life for everyone.

Second, community managers should promote the construction of community leisure environments. High-quality leisure environments can lead to high-quality community leisure behaviors and improve the image of the community. Moreover, high-quality leisure environments are conducive to the establishment and improvement of resident community identity, leisure satisfaction, and SWB [67]. Additionally, community managers should also remain attentive to resident leisure satisfaction levels to improve and enhance community leisure. Improvements in leisure satisfaction can further affect resident SWB and promote a strong community identity. Last but not least, improvements in community identity are of great significance in the long run. A strong community identity can encourage residents to participate more actively in community construction and development. In addition, improvements in community identity can positively affect resident SWB and the development of community leisure.

Third, policymakers should do their part and actively promulgate policies and regulations that can regulate people’s behavior. On one hand, they should publicize the positive effects of community leisure and provide convenient ways for residents to participate in community leisure. On the other hand, they should restrain and standardize community manager behaviors to ensure high-quality leisure activities can be provided. Policymakers must effectively coordinate and balance different concerns and use their initiative to identify and solve problems to protect the orderly development of community leisure activities.

## 6. Limitations

This research had several limitations that demand further research. First, a cross-sectional design was used in this study. As time goes on, the pandemic influence will likely evolve and resident perceptions of community leisure may change. Therefore, it is necessary to conduct a diachronic follow-up study in the future. Second, the questionnaires were mainly distributed to individuals in Beijing. Therefore, the results may not be generalizable to other countries, or even all of China. Future studies should expand the sample area to obtain more data and compare the results across different regions.

## 7. Conclusions

By integrating previous research findings and the SOR theory, this study proposed and empirically verified a conceptual model of community leisure and SWB. The main conclusions are as follows: (1) higher levels of pandemic influence and leisure environment increased leisure satisfaction; (2) pandemic influence, leisure environment, and leisure satisfaction had a positive effect on com-munity identity; (3) pandemic influence, leisure satisfaction, and community identity also combined with leisure environment and leisure time to positively influence subjective well-being; and (4) leisure satisfaction and community identity mediated the impacts of pandemic influence and leisure environment on subjective well-being.

## Figures and Tables

**Table 1:** The questionnaire items.

Dimension	Item	Source
Pandemic Influence	PI1: It makes me pay more attention to abundant leisure time.	Wenham et al. (2020) [47] Wong et al. (2016) [48]
	PI2: It makes me pay more attention to rich leisure activities.	
	PI3: It makes me pay more attention to a safe leisure environment.	
	PI4: It makes me pay more attention to a harmonious leisure atmosphere.	
	PI5: It makes me pay more attention to healthy leisure experiences.	
	PI6: It makes me pay more attention to new types of leisure activities.	
	PI7: It makes me pay more attention to community leisure.	
Leisure Environment	LE1: I have a wide variety of leisure places.	Burmeister et al. (2018) [49] Pereira et al. (2018) [50]
	LE2: I have well-equipped leisure places.	
	LE3: I have comfortable leisure places.	
	LE4: My community leisure place has a strong leisure atmosphere.	
	LE5: My community leisure activity place is civilized and orderly.	
Leisure Satisfaction	LS1: I’m very satisfied with my community leisure activities.	Wang (2017) [51] Dai et al. (2019) [52]
	LS2: Community leisure activities put me in a good mood.	
	LS3: Community leisure activities keep me fit.	
	LS4: Community leisure activities give me a sense of achievement.	
	LS5: Community leisure activities help me to improve my skills.	
	LS6: Community leisure activities help me to make friends.	
Community Identity	CI1: I really like my current community.	Thongpanya (2018) [53] Bacon and Kaya (2018) [54]
	CI2: I feel like I’m part of the community.	
	CI3: I’m very concerned about community activities.	
	CI4: I think community is important to me.	
	CI5: I identify with the educational resources provided by the community.	
	CI6: I identify with the employment conditions provided by the community.	
	CI7: I identify with the environment of the community.	
Subjective Well-Being	SW1: I’m very satisfied with my life now.	Etxeberria et al. (2019) [55] Sumargo and Novalia (2018) [56]
	SW2: I often feel happy.	
	SW3: I’m rarely bothered by negative emotions.	
	SW4: I’m satisfied with my quality of life.	
	SW5: I’ve got what I want most in life.	
	SW6: I’m satisfied with my life in general.	

**Table 2:** The demographic information of the participants.

Characteristic	Groups	*N*	%
Gender	Male	413	39.7
	Female	628	60.3
Age	Under 20 Years	36	2.9
	20–30 Years	244	19.5
	30–40 Years	347	27.7
	40–50 Years	35	2.8
	50–60 Years	243	19.4
	60 Years and Above	347	27.7
Occupation	Student	133	15.7
	Civil Servant	56	6.6
	Enterprise Staff	306	36.0
	Teachers and Technical Personnel	93	11.0
	Business Person	36	4.2
	Farmer	28	3.3
	Freelancer	69	8.1
	Retiree	88	10.4
	Unemployed	20	2.4
	Other	20	2.4
Education	Junior High School or Below	38	3.7
	High School	91	8.7
	Technical Secondary School	175	16.8
	Bachelor’s Degree	444	42.7
	Master’s Degree or Above	293	28.1
Community Type	Commercial Residential Area	506	48.6
	Protected Housing Residential Area	205	19.7
	Old Neighborhood	245	23.5
	Unit-based Community	85	8.2
Length of Residence	3 Years or Less	288	27.7
	3–5 Years	204	19.6
	5–10 Years	222	21.3
	10–15 Years	179	17.2
	Over 15 Years	148	14.2
Location	Fangshan District	31	3.0
	Huairou District	60	5.8
	Shunyi District	274	26.3
	Shijingshan District	15	1.4
	Tongzhou District	63	6.1
	Changping District	50	4.8
	Fengtai District	134	12.9
	Chaoyang District	166	15.9
	Daxing District	33	3.2
	Yanqing District	16	1.5
	Dongcheng District	34	3.3
	Xicheng District	35	3.4
	Haidian District	108	10.4
	Other	22	2.1

**Table 3:** The validity and reliability of the constructs.

Construct	Items	VIF	Loading	AVE	Composite Reliability	Cronbach’s α
Pandemic Influence	PI1	3.084	0.656	0.698	0.942	0.928
	PI2	3.483	0.705			
	PI3	3.149	0.706			
	PI4	3.771	0.779			
	PI5	2.983	0.706			
	PI6	2.980	0.766			
	PI7	2.676	0.701			
Leisure Environment	LE1	3.294	0.781	0.800	0.960	0.950
	LE2	4.435	0.855			
	LE3	4.213	0.834			
	LE4	4.394	0.845			
	LE5	2.524	0.666			
Leisure Satisfaction	LS1	3.162	0.727	0.809	0.955	0.941
	LS2	5.933	0.837			
	LS3	5.697	0.835			
	LS4	4.945	0.833			
	LS5	4.257	0.789			
	LS6	2.668	0.708			
Community Identity	CI1	2.473	0.622	0.681	0.937	0.922
	CI2	3.250	0.749			
	CI3	3.063	0.689			
	CI4	2.732	0.641			
	CI5	3.488	0.694			
	CI6	3.259	0.658			
	CI7	2.874	0.704			
Subjective Well-Being	SW1	3.519	0.728	0.698	0.933	0.913
	SW2	3.352	0.705			
	SW3	1.830	0.530			
	SW4	3.740	0.812			
	SW5	3.085	0.740			
	SW6	3.473	0.761			

**Table 4:** The discriminant validity of the constructs, according to the Fornell–Larcker criterion.

Construct	PI	LS	LE	CI	SWB
PI	**0.836 ^a^**				
LS	0.495	**0.900**			
LE	0.587	0.745	**0.894**		
CI	0.498	0.335	0.466	**0.835**	
SWB	0.637	0.663	0.721	0.582	**0.825**

^a^ The off-diagonal values are the correlations between the latent constructs and the diagonal values (indicated in bold) are the square values of the AVE values; PI, pandemic influence; LS, leisure satisfaction; LE, leisure environment; CI, community identity; SWB, subjective well-being.

**Table 5:** The discriminant validity of the constructs, according to the Heterotrait–Monotrait ratio.

Construct	SWB	LE	LS	PI	CI
SWB					
LE	0.526				
LS	0.620	0.786			
PI	0.538	0.356	0.494		
CI	0.690	0.709	0.768	0.626	

Note: The criterion for HTMT was below 0.85. PI, pandemic influence; LS, leisure satisfaction; LE, leisure environment; CI, community identity; SWB, subjective well-being.

**Table 6:** The structural relationships and hypothesis testing.

Hypothesis	Path	Beta	Standard Error	T-Statistics	Decision
H1	PI→LS	0.245	0.025	9.960 *	Supported
H2	LE→LS	0.662	0.024	27.650 *	Supported
H3	PI→CI	0.320	0.029	10.971 *	Supported
H4	LE→CI	0.291	0.031	9.953 *	Supported
H5	LS→CI	0.355	0.033	10.876 *	Supported
H6	PI→SWB	0.180	0.037	4.817 *	Supported
H7	LS→SWB	0.227	0.047	4.790 *	Supported
H8	LE→SWB	0.037	0.041	0.933	Not supported
H9	CI→SWB	0.344	0.051	6.723 *	Supported

* *p* < 0.05; PI, pandemic influence; LS, leisure satisfaction; LE, leisure environment; CI, community identity; SWB, subjective well-being.

**Table 7:** The mediating results of the hypotheses.

Path	Mediating Variable	IE	Sobel Test	Total IE	VAF	Result
PI → SWB	LS	0.055	4.317	0.344	0.481	Partial Intermediary
	CI	0.110	5.732			
LE → SWB	LS	0.151	6.533	0.289	0.869	Complete Intermediary
	CI	0.101	5.459			

PI, pandemic influence; LS, leisure satisfaction; LE, leisure environment; CI, community identity; SWB, subjective well-being.

**Table 8:** The variable calibration.

Conditions and Results	Fuzzy Calibration Point		
	Full Membership	Cross-Over Point	Full Non-Membership
SWB	4.8000	3.5000	2.2000
LE	5.0000	3.2000	1.2000
LT	5.0000	3.0000	1.0000
LS	4.8000	3.2000	1.7000
CI	4.6900	3.3000	2.0000
PI	5.0000	3.9000	2.6000

PI, pandemic influence; LS, leisure satisfaction; LE, leisure environment; CI, community identity; SWB, subjective well-being.

**Table 9:** The results of the necessity analysis.

Condition Variables	High SWB		Low SWB	
	Consistency	Coverage	Consistency	Coverage
LE (T1)	0.8064	0.7683	0.6207	0.6186
~LE (T1)	0.5997	0.6018	0.7675	0.8057
LT (T2)	0.8056	0.7374	0.6378	0.6106
~LT (T2)	0.5746	0.5746	0.7257	0.7962
LS (T3)	0.8235	0.7797	0.5932	0.5875
~LS (T3)	0.5644	0.5701	0.7776	0.8217
CI (T4)	0.8191	0.8127	0.5500	0.5708
~CI (T4)	0.5674	0.5466	0.8195	0.8258

PI, pandemic influence; LS, leisure satisfaction; LE, leisure environment; CI, community identity; SWB, subjective well-being.

**Table 10:** The results of the conditional configuration analysis.

Conditional Configuration	SWB					
	1	2	3	4	5	6
LE (T1)				▉	⊗	⊗
LT (T2)				▉		
LS (T3)		●	⊗		●	
CI (T4)	▉					
PI (T5)		⊗	●			●
Consistency	0.8127	0.7893	0.7952	0.8316	0.8015	0.7963
Raw Coverage	0.8191	0.5586	0.4150	0.7120	0.4806	0.4526
Unique Coverage	0.0678	0.0083	0.0011	0.0128	0.0005	0.0007
Solution Consistency	0.7270					
Solution Voverage	0.9210					

▉ or ● indicate the presence of a condition; □ or ⊗ indicate the absence of a condition; ▉ or □ indicate a core condition; ● or ⊗ indicate a peripheral condition; blank spaces indicate a “don’t care” condition; PI, pandemic influence; LS, leisure satisfaction; LE, leisure environment; CI, community identity; SWB, subjective well-being.

## Data Availability

Not applicable.
